# Inflammatory markers in palatine tonsils of children with obstructive sleep apnea syndrome^☆^

**DOI:** 10.1016/j.bjorl.2018.08.001

**Published:** 2018-08-31

**Authors:** Vitor Guo Chen, Viviane Maria Guerreiro da Fonseca, Jônatas Bussador Amaral, Cíntia Meirelles Camargo-Kosugi, Gustavo Moreira, Eduardo Macoto Kosugi, Reginaldo Raimundo Fujita

**Affiliations:** a Universidade Federal de São Paulo (UNIFESP), Escola Paulista de Medicina (EPM), Departamento de Otorrinolaringologia e Cirurgia de Cabeça e Pescoço, São Paulo, SP, Brazil; b Universidade Federal de São Paulo (UNIFESP), Escola Paulista de Medicina (EPM), Departamento de Otorrinolaringologia e Cirurgia de Cabeça e Pescoço – Centro de Pesquisa Translacional de Otorrinolaringologia e Cirurgia de Cabeça e Pescoço, São Paulo, SP, Brazil; c Universidade Federal de São Paulo (UNIFESP), Escola Paulista de Medicina (EPM), Instituto do Sono, São Paulo, SP, Brazil

**Keywords:** Inflammation mediators, Obstructive sleep apnea, Palatine tonsil, Child, Mediadores de inflamação, Apneia obstrutiva do sono, Tonsila palatina, Criança

## Abstract

**Introduction:**

Obstrutive sleep apnea syndrome is characterized by repeated episodes of upper airway obstruction, associated with intermittent hypoxia and hypercapnia, and the main risk factor in childhood is adenotonsillar hypertrophy. The lymphocytes in these structures are responsible for local and systemic immune responses.

**Objective:**

Verify the levels of the inflammatory markers, IL-1β, IL-4, IL-6, IL-8, IL-10, IL-15, TNF-α, CRP and α1-GP, in the tonsils of children with and without obstructive sleep apnea syndrome.

**Methods:**

This cross-sectional prospective study included 34 children with complains of snoring, difficulty breathing during sleep or recurrent tonsillitis. Patients underwent to a complete otorhinolaryngological examination, nasal endoscopy and polysomnography and were divided into two groups with 17 children each: obstructive sleep apnea syndrome group and control group. All underwent an adenotonsillectomy. Cytokines were measured in the collected tonsils (ELISA and Multiplex methods).

**Results:**

Statistically significant increasing were observed between IL-8 and IL-10 cytokines of patients with obstructive sleep apnea when compared to the control group; also between c-reactive protein and α1-GP of the tonsils cortical region in children with obstructive sleep apnea syndrome when compared with the medullary region. There were no statistically significant differences for the remaining inflammatory mediators.

**Conclusion:**

After the analysis of the levels of pro and anti-inflammatory markers (IL-1β, IL-4, IL-6, IL-8, IL-10, Il-15, TNF-α, CRP, α1-GP) in the tonsils, we observed higher levels of markers IL-8 and IL-10 in pediatric patients with obstructive sleep apnea syndrome.

## Resumo

**Introdução:**

A síndrome da apneia obstrutiva do sono é caracterizada por episódios repetidos de obstrução das vias aéreas superiores, associados a hipóxia intermitente e hipercapnia, e o principal fator de risco na infância é a hipertrofia adenotonsilar. Os linfócitos nessas estruturas são responsáveis por respostas imunes locais e sistêmicas.

**Objetivo:**

Dosar os marcadores inflamatórios, IL-1β, IL-4, IL-6, IL-8, IL-10, IL-15, TNF-α, PCR e α1-GP, nas tonsilas de crianças com e sem síndrome da apneia obstrutiva do sono.

**Método:**

Estudamos prospectivamente 34 crianças que se queixavam de ronco, dificuldade para respirar durante o sono ou tonsilites recorrentes. Os pacientes foram submetidos a exame otorrinolaringológico completo, endoscopia nasal e polissonografia e foram divididos em dois grupos com 17 crianças cada: síndrome de apneia obstrutiva do sono e controle. Todos foram submetidos à adenotonsilectomia. As citocinas foram medidas nas tonsilas coletadas (métodos ELISA e Multiplex).

**Resultados:**

Com diferenças estatisticamente significantes, observou-se aumento das citocinas IL-8 e IL-10 em pacientes com apneia obstrutiva do sono em comparação ao grupo controle, assim como aumento dos níveis de proteína C reativa e de α1-GP na região cortical das tonsilas de crianças portadoras de síndrome da apneia obstrutiva do sono em comparação com a região medular. Não houve diferenças estatisticamente significantes para o restante dos mediadores inflamatórios.

**Conclusão:**

Após a análise dos níveis de marcadores pró e anti-inflamatórios (IL-1β, IL-4, IL-6, IL-8, IL-10, Il-15, TNF-α, PCR, α1-GP) nas tonsilas, observamos níveis mais altos de marcadores IL-8 e IL-10 em pacientes pediátricos com síndrome da apneia obstrutiva do sono.

## Introduction

Obstructive sleep apnea syndrome (OSAS) is one of the sleep-disordered breathing (SDB) defined as repeated episodes of upper airway obstruction, associated with hypoxia and intermittent hypercapnia.^1^ SDB includes primary snoring, upper airway resistance syndrome and, as the most important condition, OSAS.^2^ SDB is very common in childhood and it is estimated that 3%–26% of young children have habitual snoring and 1.2%–5.7% have OSAS.^3^, ^4^, ^5^

It is suggested that OSAS can induce a systemic pro-inflammatory response and its magnitude can be involved in the final morbidity.^6^ Alberti et al.^7^ measured the levels of the pro-inflammatory cytokines IL-1β, IL-6 and TNF-α and anti-inflammatory cytokines IL-10 and TGF-β in patients with OSAS. An increase in TNF-α and IL-6 was observed, when compared with the control group. Recently, it has been shown that the spontaneous TNF-α production by monocytes is increased in adult patients with moderate or severe OSAS and that it regresses after treatment with Continuous Positive Air Pressure (CPAP).^8^ Gozal et al.^9^ demonstrated that surgical treatment of OSAS (a standard treatment for the disease in children) resulted in significant reductions in TNF-α levels, with reciprocal sleep latency prolongation. Furthermore, it has been shown that C-Reative Protein (CRP) is related to OSAS-mediated cognitive morbidity.^10^ The increase in serum levels of CRP, IL-6 and TNF-α are important risk factors for atherosclerosis, cerebrovascular accident (stroke) and cardiovascular diseases,^11^ and their levels are increased in OSAS.^12^, ^13^, ^14^ It is possible that OSAS affects not only the pro-inflammatory cytokines, but also blocks the expression of anti-inflammatory cytokines, such as IL-10, that inhibits a wide range of pro-inflammatory responses, including those affecting the vessel wall.^15^, ^16^, ^17^

The main risk factors for OSAS in childhood is adenotonsillar hypertrophy, obesity, neuromuscular diseases and craniofacial alterations.^18^ Beyond these factors, palatine and pharyngeal tonsil hypertrophy stand out as the main etiologies.^19^, ^20^, ^21^ The exact mechanism underlying follicular lymphoid proliferation and hyperplasia remains poorly understood. In adults, there are several lines of evidence suggesting that local and systemic upper airway inflammation entails the pathophysiology of upper airway mechanical dysfunction. It has been shown that the number of immune cells is significantly higher in the mucosa and muscle of adults with OSAS.^22^ Similar increases in systemic and regional inflammatory markers have also been reported in children with OSAS.^10^ Therefore, it is assumed that cell proliferation in the palatine tonsil tissue of children with OSAS is different from that in children with recurrent tonsillitis, possibly reflecting the pathological mechanisms and types of cell involved in upper airway lymphoid tissue proliferation in these two situations.^23^ Considering that, the aim of the present study was to assess OSAS’ impact on inflammatory pattern of palatine tonsils of children.

## Methods

### Study population

Thirty-four patients assisted at a Pediatric Otorhinolaryngology Clinic aged 3–12 years of both genders, who underwent tonsillectomy from October 2013 to June 2014 for OSAS, primary snoring or recurrent tonsillitis treatment, were prospectively evaluated.

OSAS was considered present when polysomnography parameters demonstrated obstructive Apnea Index (AI) ≥ 1 event per hour or Apnea-Hypopnea Index (AHI) ≥ 1.5 events per hour and minimal oxygen saturation (SpO_2_ nadir) ≤ 92%. So, absence of OSAS was considered when polysomnography parameters demonstrated AI < 1 event per hour or AHI < 1.5 events per hour and SpO_2_ nadir > 92%. Primary snoring was considered in the presence of habitual snoring with the absence of OSAS confirmed by polysomnography. And recurrent tonsillitis was considered by the Paradise's criteria^24^ in the absence of OSAS confirmed by polysomnography.

Inclusion criteria:

- Age below 12 years;
- Surgical indication for tonsillectomy due to OSAS, primary snoring or recurrent tonsillitis;
- Tonsillectomy performed between October 2013 and June 2014;
- Children or parents/guardians who accepted to participate in the study.

Exclusion criteria:

- Established or under investigation syndrome with orofacial alterations;
- Pulmonary or cardiac diseases and obesity;
- Established or under investigation diseases of metabolic or myopathic origin;
- Upper airway infection at the time of surgery;
- Immunodeficiency;
- Previous tonsillectomy;
- Did not understand the initial instructions;
- Did not undergo the assessments in predetermined periods according to study protocol.

### Study design

This study was approved by the Institutional Review Board under protocol number 97,863 and written informed consent was obtained from all children's parents and/or guardians.

It was a cross-sectional study based on two groups: OSAS group, formed by 17 children with OSAS and without recurrent tonsillitis submitted to tonsillectomy; and Control group, formed by 17 children without OSAS submitted to tonsillectomy due to primary snoring and/or recurrent tonsillitis.

After inclusion and exclusion criteria were considered, children were submitted to bilateral tonsillectomy (with or without adenoidectomy) under general anesthesia in an operating room of university's hospital. Palatine tonsils were removed together with their capsules by cold-knife surgery, briefly stored in a container with dry ice at −80 °C and carried to the lab to be processed. Palatine tonsils were sectioned into two parts, cortical and medullary zones and the following inflammatory proteins concentration were measured: Alpha-1 acid glycoprotein (α1-GP), C-Reative Protein (CRP), Interleukin (IL)-1β, IL-4, IL-6, IL-8, IL-10, IL-15 and tumor necrosis factor (TNF)-α.

Inflammatory mediators’ concentrations were compared between cortical and medullary zones for each group, and then total concentrations of each group were compared.

### Immunological analysis

The palatine tonsil samples were sectioned into two parts, cortical and medullary. Subsequently, the tissue was mechanically dissociated (TissueRuptor, Qiagen, Hilden, Germany) in an Eppendorf tube containing 400 μL of RPMI 1640 culture medium (Sigma–Aldrich, St. Louis, MO, USA). Subsequently, the fragments were centrifuged for 5 min at 20.817 × *g* speed. After protein extract separation (supernatant phase), protein sample quantification was performed using Pierce's BCA Protein Assay Reagent Kit (Thermo Fisher Scientific, Waltham, MA, USA) as described below.

A standard curve was performed using BSA (Bovine Serum Albumin) standard for each point of the curve, in serial dilutions. For quantification, 2 mL of Working Reagent (WR) were used in each Eppendorf tube. The WR mix was prepared as follows: 50 parts of BCA Reagent A and 1 part of BCA Reagent B (50:1 Reagent A: B). The tubes were incubated for 30 min at 37 °C in the dark and were subsequently read in a spectrophotometer (NanoDrop 2000 Thermo Fisher Scientific, Waltham, MA, USA) at a wavelength of 562 nm.

Aiming to equate the assessed protein concentration in all samples, it was standardized that all were at a concentration of 3000 μg/mL.

After being subsequently placed in 2 mL conical tubes, these samples were stored at −80 °C. α1-GP was analyzed by ELISA reading kit (USCN Life Science), CRP by Milliplex-Human CVD Panel 3 reading kit (MERK®), and IL-1β, IL-4, IL-6, IL-8, IL-10, IL-15 and TNF-α by Milliplex-Human Cytokine/Chemokine reading kit (MERK®), following the procedures and recommendations of each kit manufacturer.

### Statistical analysis

Normal distribution of data was verified by Kolmogorov–Smirnov test. The comparison between inflammatory mediators found in the cortical and medullary zones of each group was performed by Student's *t*-test for dependent samples or the Wilcoxon test, depending on the presence or absence of normality in data distribution, respectively. Comparison of inflammatory mediators between groups was performed by Student's *t*-test for independent samples or the Mann–Whitney *U*-test, also depending on the normality of data distribution.

For all statistical analysis, *p*-values below 5% were considered significant.

## Results

Thirty-four patients were included in this study: 17 children in the OSAS group and 17 children in the control group. Fifteen patients were males. Mean age was 7.55 years old and median of 8.5 years.

Only CRP and α1-GP were significantly differently expressed between cortical and medullary zones in the OSAS Group. The cortical zone showed higher levels of these proteins (Table 1).

**Table 1:** Comparison between values of inflammatory mediators in the cortical and medullary zone of palatine tonsils from OSAS Group expressed in pg/mL.

Mediator	Palatine tonsils OSAS group				*p*
	Cortical		Medullary		
	Mean	SD	Mean	SD	
IL-1ß	99.58	113.78	104.49	115.85	0.62
IL-4	6.16	1.58	7.17	2.92	0.20
IL-6	6.68	4.49	6.63	3.38	0.97
IL-8	1,009.27	881.07	1,168.13	1,151.96	0.42
IL-10	13.74	12.64	12.42	11.21	0.55
IL-15	3.80	0.84	3.92	1.39	0.54
TNF-α	47.66	22.53	53.13	29.30	0.24
CRP	15.68	18.41	8.84	11.06	0.04
α1-GP	10.48	9.92	7.00	6.22	0.05

IL, interleukin; TNF, tumor necrosis factor; CRP, C-reactive protein; α1-GP, alpha1-glycoprotein; SD, standard deviation; OSAS, obstructive sleep apnea syndrome; pg/mL, picogram per milliliter.

In the Control Group, only IL-1β was differently expressed, presenting higher levels in the medullary zone (Table 2).

**Table 2:** Comparison between values of inflammatory mediators in the cortical and medullary zone of palatine tonsils of the Control Group, expressed in pg/mL.

Mediator	Palatine tonsils control group				*p*
	Cortical		Medullary		
	Mean	SD	Mean	SD	
IL-1ß	60.21	44.82	134.30	187.74	0.02
IL-4	6.36	3.39	6.62	3.29	0.88
IL-6	7.22	5.62	7.23	4.38	0.60
IL-8	665.22	630.84	663.67	509.48	0.30
IL-10	9.05	8.63	12.34	16.29	0.41
IL-15	3.90	1.02	4.03	1.45	0.49
TNF-α	43.03	14.73	45.79	13.02	0.58
CRP	12.04	11.00	13.87	15.56	0.46
α1-GP	10.98	9.33	11.31	12.07	0.81

IL, interleukin; TNF, tumor necrosis factor; CRP, C-reactive protein; α1-GP, alpha1-glycoprotein; SD, standard deviation; OSAS, obstructive sleep apnea syndrome; pg/mL, picogram per milliliter.

When comparing the total inflammatory mediators’ concentration between the OSAS and Control Group, the OSAS Group presented significantly higher levels of IL-8 and IL-10 than the Control Group (Table 3).

**Table 3:** Comparison between values of total inflammatory mediators of the palatine tonsils of patients with and without OSAS, expressed in pg/mL.

Mediator	Palatine tonsils				*p*
	OSAS group		Control group		
	Mean	SD	Mean	SD	
IL-1ß	102.03	112.98	97.26	139.67	0.34
IL-4	6.67	2.37	6.49	3.29	0.20
IL-6	6.66	3.91	7.22	4.96	0.32
IL-8	1,088.77	1,012.05	664.44	565.13	0.04
IL-10	13.08	11.77	10.69	12.95	0.04
IL-15	3.86	1.13	3.96	1.24	0.46
TNF-α	50.39	25.86	44.41	13.77	0.41
PCR	12.26	15.34	12.96	13.32	0.30
α1-GP	8.74	8.33	11.15	10.63	0.21

IL, interleukin; TNF, tumor necrosis factor; CRP, C-reactive protein; α1-GP, alpha1-glycoprotein; SD, standard deviation; OSAS, obstructive sleep apnea syndrome; pg/mL, picogram per milliliter.

Statistically significant differences were found for the levels of IL-8 and IL-10 mediators.

## Discussion

Adenotonsillar hypertrophy is the main pathophysiological mechanism underlying obstructive sleep apnea syndrome (OSAS) in children. The literature shows that there is increased expression of several inflammatory response mediators in the tonsils of patients with OSAS.^7^, ^11^, ^23^ This idea emphasizes the hypothesis that the increase in local inflammation in children with sleep apnea can promote tonsil proliferation.^23^

In the present dissertation, it was observed that the levels of inflammatory mediators IL-1β, IL-4, IL-6, IL-15, TNF-α, CRP and α1-GP in the tonsils of children with OSAS showed no significant difference when compared to the group without OSAS. Nevertheless, a significant increase was observed in IL-8 and IL-10 levels in patients with OSAS, when compared with the control group. Similar to what was described, other authors reported that elevated levels of IL-8, a chemokine that plays a key role in neutrophil and monocyte adhesion to the vascular endothelium, have been demonstrated in patients with OSAS.^13^ Agren et al.^25^ found an increased production of IL-8 and IL-10 in hypertrophic tonsils of children with OSAS. However, this finding also occurred in the tonsils of children with recurrent tonsillitis, even without clinical signs of infection. Furthermore, they observed an increase in other cytokines, namely IL-2, IL-4, IL-6, TNF-α, TNF-β and IFN-γ.

Other results, different from those found in this study, have been described in the literature. Lindberg et al.^26^ assessed the markers IL-1α, IL-1β, IL-2, IL-4, IL-6, IL-8, IL-10, TNF-α and TNF-β in the suspension of hypertrophic tonsil cells with severe OSAS with no history of infection *vs*. tonsils with recurrent infections. Levels of IL-1β, IL-2 and IL-6 showed a significant increase in the recurrent tonsillitis group when compared to the hypertrophy group. Yokoe^27^ reported that IL-6 and CRP levels were elevated in patients with OSAS when compared to an obese control group, showing there was a reduction in these levels after treatment with CPAP. It was suggested that OSAS systemic inflammation could exist regardless of obesity. Andersson et al.^28^ demonstrated an increased production of 19 inflammatory markers (including IL-4, IL-6 and IL-8) in human tonsils with chronic infection and found different cytokine patterns in recurrent tonsillitis group when compared with the infectious mononucleosis group. They rarely observed IL1β and TNF-α in the recurrent tonsillitis group. On the other hand, other authors identified high concentrations of IL-1β, IL-6 and TNF-α in adenotonsillar tissues, believing it to be an expression of local overproduction due to monocyte-macrophage activation resulting from repeated stimuli from pathogen agents.^29^ Kim et al.^23^ observed that both the expression and the release of pro-inflammatory cytokines, such as IL-1β, IL-6 e TNF-α, were increased in cell cultures of children with OSAS.

Even though these studies show changes in the levels of many cytokines studied in this dissertation, it is not possible to identify a consensus regarding the difference between patients with OSAS and patients with recurrent tonsillitis. A condition that might have contributed for the fact that the majority of the assessed cytokines did not show any significant difference between the groups (case and control) was the possibility that both apnea and recurrent infection alter inflammatory marker levels. Perhaps a study comparing these groups in children without OSAS, recurrent tonsillitis or even obstructive tonsils can answer this question.

Taking the present results in consideration – IL-8 and IL-10 predominance in palatine tonsils of OSAS patients – one may deduce that palatine tonsils do not seem to be the determinant factor in the inflammatory pathophysiology of OSAS, especially considering that IL-10 is an anti-inflammatory cytokine.

Another important factor to be discussed is the comparison of inflammatory marker measurements between the cortical and medullary regions of the tonsils. CRP and α1-GP levels were higher in the cortical region of the tonsils of patients with sleep apnea when compared to the medullary region, a result not observed in tonsils of children without OSAS. Increased CRP serum levels in children with OSAS would be evidence of the presence of a systemic inflammatory process, which could also contribute to increased proliferation of upper airway lymphoid tissue.^30^ This difference demonstrated between the cortical and medullary regions may be related to this proliferation.

Some studies on OSAS and cytokines differentiate the groups according to the Apnea and Hypopnea Index (AHI) or even the degree of sleep apnea. A study carried out in 2012 associated the elevated CRP levels in patients with sleep apnea with its severity, regardless of obesity.^30^ Many studies have associated increased TNF-α serum levels with OSAS severity.^31^ Others have demonstrated that the increase in TNF-α and IL-6 levels were accompanied by an increase in the AHI in patients with sleep apnea.^9^ However, Chu and Li^32^ concluded that the increases in TNF-α and IL-6 are mainly influenced by the obesity factor. The present study excluded all obese children or those with any metabolic/endocrine diseases, but OSAS severity was not consider.

Another bias is that allergic sensitization was not specified. Previous studies have reported a high prevalence of allergy in children with OSAS.^33^, ^34^ Moreover, the use of topical corticosteroids in some patients may have altered cytokines levels, although the bioavailability of most nasal corticosteroids is very low.

Lastly, only palatine tonsils were included in the present study, but pharyngeal tonsils also play an important role in the pathophysiology of OSAS in pediatric patients and may be associated with the inflammatory markers involved in sleep disorders. Therefore, future and more specific studies will need to evaluate these issues and clarify the role of cytokines in OSAS *versus* recurrent tonsillitis.

## Conclusion

Palatine tonsils of patients with OSAS showed higher expression of CRP and α1-GP in cortical than in medullary zone, whereas palatine tonsils of patients without OSAS showed higher expression of IL-1β in medullary than in cortical zone. OSAS promoted different pattern of inflammation in palatine tonsils, with predominance of IL-8 and IL-10 when compared to controls. It is not possible yet to identify a consensus regarding the difference between patients with OSAS and patients with recurrent tonsillitis.

## Conflicts of interest

The authors declare no conflicts of interest.
